# Deep learning-based beat-to-beat delineation of heart sounds and fiducial points in seismocardiography

**DOI:** 10.3389/fdgth.2025.1699611

**Published:** 2025-12-04

**Authors:** Emil Korsgaard, Ahmad Agam, Peter Søgaard, Kasper Janus Grønn Emerek, Kasper Sørensen, Jørn Wulff Helge, Johannes Jan Struijk, Samuel Emil Schmidt

**Affiliations:** 1 Department of Health Science and Technology, Aalborg University, Aalborg, Denmark; 2 Department of Cardiology, Aalborg University Hospital, Aalborg, Denmark; 3 Department of Clinical Medicine, Aalborg University, Aalborg, Denmark; 4 VentriJect ApS, Hellerup, Denmark; 5 Department of Biomedical Sciences, University of Copenhagen, Copenhagen, Denmark

**Keywords:** seismocardiography, SCG, deep learning, segmentation, U-Net

## Abstract

**Introduction:**

The application of deep learning methods in automatic delineation of fiducial points in seismocardiography (SCG) on a beat-to-beat basis provides the possibility of obtaining a novel and comprehensive approach to assess and monitor myocardial mechanics and hemodynamic status. Therefore, the aim of this study was to develop an adaptive and data-driven algorithm for automatic delineation of 11 fiducial points in SCG.

**Methods:**

SCG signals from subjects both with and without known cardiac disease (CD) were included. A semi-automatic annotation pipeline was prepared for effective annotation of fiducial points for each individual cardiac cycle, in which 42,452 individual beats from 198 subjects were annotated. A deep learning model with U-Net architecture was developed to detect 11 fiducial points and predict multiple time intervals in the SCG signal. The evaluation metrics were positive predictive value and sensitivity.

**Results:**

The median positive predictive value and sensitivity of the algorithm ranged between 0.809 and 1.000 and 0.843 and 0.918 for different fiducial points, respectively.

**Conclusion:**

A novel algorithm for automatic detection of 11 fiducial points in SCG was developed and tested in subjects both with and without CD.

## 1 Introduction

Seismocardiography (SCG) is a non-invasive and easy-to-use technique for measuring the low-frequency vibrations in the chest wall that are produced by the mechanical activity of the heart during the cardiac cycle. SCG was first introduced in 1957 by Mounsey (1). After a long period of stagnation within SCG research, the introduction of micro-electromechanical system accelerometer technology in recent decades has facilitated easy SCG acquisition, which has improved and reignited the field (2).

The clinical applicability of SCG critically depends on the detection of fiducial points and on understanding the meaning of these fiducial points in terms of physiological events in the cardiac cycle. In recent years, fiducial points have been defined by correlating waveforms and peaks in the SCG with cardiac events such as aortic valve opening, aortic valve closure, mitral valve opening, mitral valve closure, peak systolic outflow (PSO), and peak atrial inflow (3–6). Thus, SCG also has the potential to measure left ventricular ejection time, isovolumetric contraction time, and isovolumetric relaxation time. Additionally, a correlation between SCG-derived parameters and preload, pulmonary artery pressure, and electromechanical activation time has been shown (7–11), and thus SCG can be used to assess biventricular pacing and the clinical status of heart failure, and estimate cardiorespiratory fitness (12–15). Moreover, the detection of the fiducial points on a beat-to-beat basis would enable the rapid assessment of changes in cardiac mechanics and hemodynamic status, making SCG suitable for the continuous monitoring of cardiac patients. However, SCG signal quality varies significantly depending on factors such as noise due to patient movement or talking, respiration, heart rhythm variations, anatomical variations, artifacts, and pathophysiology. All these factors complicate the development of automatic algorithms.

Various types of algorithms for detecting fiducial points in SCG have been suggested in the literature, and some of these use guiding signals, such as electrocardiography (ECG) or photoplethysmography, in which known reference points are used to detect the fiducial points in SCG (16, 17). This requires additional and thus more complicated hardware compared with a standalone SCG method.

Multiple algorithms based only on SCG have also been suggested. These algorithms rely on predefined decision rules, thresholds, time intervals, etc., and have shown promising results (16, 18–23). However, the decision rules, thresholds, etc., are challenging to define generally and be representative of different cases, since the SCG signal exhibits high variability in amplitude, timing, and noise level across anatomical variations, different accelerometers, and cardiac diseases (CDs). Therefore, these algorithms require extensive adaptation for different use cases. Consequently, it is challenging to define a general algorithm for fiducial point detection. In addition, the evaluation of such algorithms has often been based on a low number of subjects, restricting the representativeness of such an algorithm to also perform well on a more diverse dataset.

An alternative approach is the use of deep learning algorithms for the semantic segmentation of SCG signals. An advantage of this approach is that such models are data-driven and provide the possibility to quickly retrain the algorithm based on different CDs, anatomical variations, ages, etc., to extend the model's application area.

The use of deep learning for the semantic segmentation of heart sounds in phonocardiography (PCG) in subjects both with and without known CDs (24) and detecting heartbeats in SCG (25) has shown promising results. However, these algorithms have been trained and validated on a limited amount of data and only detect the location of heart sounds. No algorithms that use deep learning for semantic segmentation have been proposed for detecting multiple fiducial points in SCG on a beat-to-beat basis.

The identification of multiple fiducial points in the ECG on a beat-to-beat basis using deep learning and simple postprocessing methods has been proposed in subjects with different cardiac diseases (26). Since SCG and ECG are similar in rhythmicity, the method also has potential in SCG.

Therefore, the aim of this study was to develop a deep learning-based algorithm for automatic beat-to-beat detection of multiple fiducial points in SCG in healthy subjects and subjects with cardiac diseases.

## 2 Materials and methods

In this study, data from SCG and simultaneously measured lead I and lead II ECG from 212 subjects in six previously published studies were gathered and used for algorithm development and evaluation. The concurrent ECG data were only used to guide the manual annotation of the SCG signal. A U-Net model was used to transform the SCG data into 19 segmentation maps (SMs), which, through postprocessing, were converted to times of occurrence of fiducial points. These steps together constitute the SeismoTracker algorithm.

### 2.1 Subjects

Data from six studies (6, 10–12, 14, 27) were gathered into a single database for this study. The data were collected at Aalborg University and Copenhagen University. In the dataset, SCG and concurrent lead I and lead II ECG were obtained with a sampling frequency of 5,000 Hz. The accelerometer was a Colibrys Model SF1600SA in one study (14) and a Silicon Designs 1521 in the remaining studies (6, 10–12, 27). In all the studies, the accelerometer was placed at the xiphoid process using double adhesive tape. In two studies (12, 27), the signals were acquired at rest in the supine position from subjects with no known CD. In another two studies (6, 10), the signals were obtained both at rest in the supine position and in the 30-degree tilt position in subjects with no known CD. In the fifth study (11), the signals were obtained at rest and after a saline infusion in subjects with and without known CD (hypertrophic cardiomyopathy, dilated cardiomyopathy, aortic valve disease, and ischemic heart disease). In the sixth study (14), the signals were obtained from subjects with a biventricular pacemaker implanted more than 3 months before the study due to heart failure and left bundle branch block and biventricular pacing more than 95% of the time. In this study, recordings were obtained with no pacing, left ventricular pacing, right ventricular pacing, and pacing of both the right and left ventricles simultaneously.

The subjects from each study were divided into training (70%), validation (10%), and test (20%) to ensure that subjects from each dataset were represented in both the development and validation phases. Consequently, no-CD and CD subjects were represented in the training, validation, and test subsets. The exclusion of subjects was performed before the split.

### 2.2 Fiducial points to detect

A large set of fiducial points was defined by Sørensen et al. (6), and 11 fiducial points were of interest to this study, namely, seven fiducial points in the systolic complex and four in the diastolic complex. These 11 fiducial points were the most consistent across the subjects and provide an adequate representation of the mechanics within a heartbeat. Even though the fiducial points were defined in a mean beat derived from the aligned beats by Sørensen et al. (6), they were processed on a beat-to-beat basis in this study.

**Figure 1:**
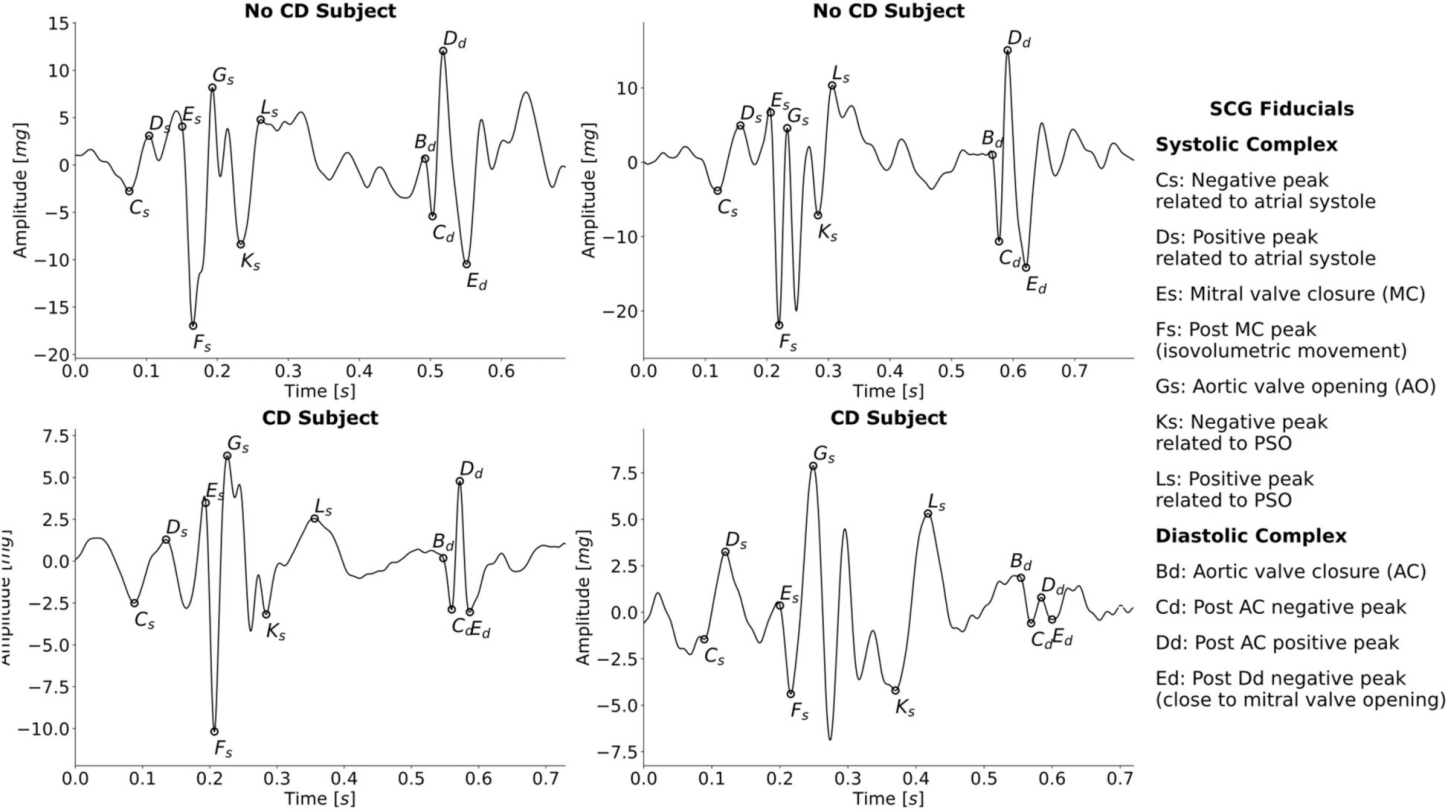
Examples of SCG beats with annotated fiducial points from four different subjects, two without CD and two with CD. Additionally, the relationship between each fiducial point and cardiac event is indicated. PSO, peak systolic outflow.

The fiducial points in the systolic complex were Cs, Ds, Es, Fs, Gs, Ks, and Ls, while the fiducials in the diastolic complex were Bd, Cd, Dd, and Ed. In Figure 1, the fiducial points in a single beat for four different subjects and the fiducial points’ relation to physiological events in the cardiac cycle are illustrated.

### 2.3 Manual data annotation prior to algorithm development

To develop a data-driven model for fiducial point detection, it is necessary to have thoroughly annotated the 11 fiducial points in each of the SCG beats manually as ground truth data. Manual annotation was performed using a tailored Python tool. The beat-to-beat manual annotations were made for the 11 fiducial points according to the rules described by Sørensen et al. (6), with concurrent ECG used to guide the manual annotation of the data. The rules followed in the manual annotation are thoroughly described in Sørensen et al. (6) and will thus not be described in this section. If at least one of the fiducial points in a given beat was not clearly identifiable, according to the rules, no fiducial points were annotated in that given beat. This was mainly due to noise in some beats.

Additionally, the R-peak in each ECG beat was also annotated to obtain the heart rate. The R-peak was initially annotated using the algorithm described by Jensen et al. (28) and adjusted by the annotator where the algorithm predicted wrong or did not predict an R-peak. The R-peak is the most positive deflection in the QRS complex. If an R-peak was not clearly identifiable, e.g., with no clearly identifiable QRS complex or no single clear positive R-peak deflection, no R-peak was annotated for the given beat. There was only one main annotator, namely, the first author, E.K., a PhD student with extensive knowledge of SCG morphology. Moreover, the rules that were followed have been thoroughly described, ensuring representativity. However, if the main annotator was in doubt about the data from some subjects, the annotation was cross-checked by the last author, S.E.S.

The 212 subjects included had recordings of between 30 s and 15 min in duration, with 11 fiducial points per SCG beat, one R-peak per ECG beat, and median heart rates ranging from 36 bpm to 94 bpm, which resulted in a very high number of annotated beats. As described by Koshrow-Kavar et al. (21), 2 years were required to manually annotate 55,164 beats with fewer fiducial points compared to this work; thus, alternative and more effective methods to annotate SCG data are required.

Consequently, the data in this study were annotated in an annotate-train loop, similar to a process that has been used to develop powerful segmentation models in biomedical image processing (29, 30). First, data from three subjects with no CD and three subjects with CD were manually annotated. A preliminary version of SeismoTracker, consisting of the same U-Net structure with random initial parameters and simple postprocessing, was trained based on the first set of manually annotated data. Then, an updated version of SeismoTracker was used to predict the initial fiducial points for five new subjects randomly selected from the dataset. Each of these initial annotation fiducial points was adjusted manually according to the annotation rules. Based on the new and previously obtained annotations, the preliminary SeismoTracker was retrained and used to initially annotate another five new subjects. This process was repeated until all 42,452 beats were annotated, within a period of 3 weeks.

The annotator was blinded to whether the given SCG signal belonged to the training, validation, or test split to reduce annotation bias. Furthermore, each initial annotation point was evaluated by the annotator according to the rules, independent of the algorithm's prediction. Moreover, to prevent data leakage, only the subjects in the predefined training set were included in the ongoing retraining process. The manual annotation followed the annotation rules of Sørensen et al. (6), and the manual annotation can thus be considered ground truth annotations, independent of the preliminary version's predictions. Thus, the preliminary version was used as a time accelerator; however, all the annotations were thoroughly evaluated according to the rules, ensuring reproducibility.

### 2.4 Preprocessing of SCG signals

Before the annotation process, the SCG signals were filtered using a forward-backward third-order low-pass Butterworth filter with a cutoff frequency of 60 Hz to smooth the signal and attenuate any high-frequency noise, thus promoting peaks that were fiducial points. This filter was followed by a forward-backward third-order high-pass Butterworth filter with a cutoff frequency of 1 Hz to remove any low-frequency noise. The SCG signals were annotated in full length.

### 2.5 Conversion from manually annotated fiducial points to ground truth segmentation maps

The annotated fiducial points were converted into 19 binary SMs with the same number of samples as the corresponding SCG in order to convert the fiducial point timings into semantic segmentation maps that U-Net models are generally suitable for predicting. Additionally, the binary SMs represented intervals between the different fiducial point timings, which added temporal and contextual information for the U-Net to recognize. The fiducial points Es and Bd were determined by their subsequent valley fiducial and were therefore not included in the semantic segmentation.

**Figure 2:**
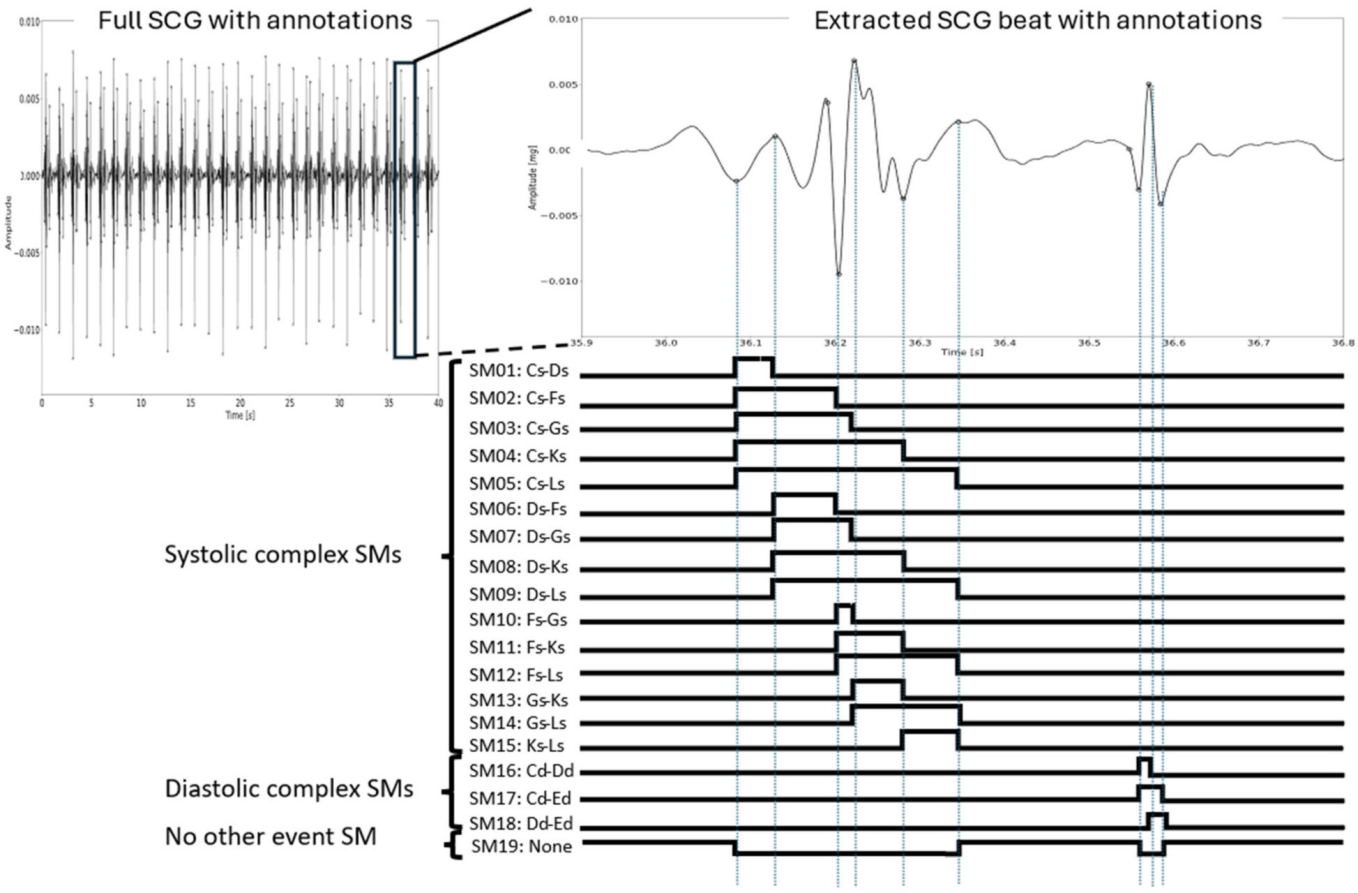
The process of generating SMs for a single heartbeat. Each combination of fiducial points within each complex was identified, thus defining the unique intervals for which the samples in the corresponding SM were set to 1. In the no-event SM (SM19), these samples were set to 1, and the corresponding samples in all the other SMs were 0. Note that the signals were not segmented into beats prior to the annotation. This is just an illustration for one beat in a sequence for convenience.

The process of converting the 11 manually annotated fiducial points to 19 SMs is illustrated in Figure 2 for one beat. The process was repeated for the entire signal. An SM was created for each unique combination of fiducial points in each complex independently, resulting in 15 SMs from the systolic complex, namely, Cs–Ds, Cs–Fs, Cs–Gs, Cs–Ks, Cs–Ls, Ds–Fs, Ds–Gs, Ds–Ks, Ds–Ls, Fs–Gs, Fs–Ks, Fs–Ls, Gs–Ks, Gs–Ls, and Ks–Ls, and three from the diastolic complex, namely, Cd–Dd, Cd–Ed, and Dd–Ed. The last SM was a no-event SM, indicating that no fiducial point interval was present.

Each SM had the same number of samples as the SCG. In each SM, the samples between the given set of fiducial points were set to 1 within a beat, while the others were set to 0, e.g., for the Cs–Ds SM, the samples within these fiducial points in the same beat were set to 1. Therefore, a sequence of consecutive samples assumed to be 1 in an SM would indicate the number of samples within the given fiducial point interval in each heartbeat. Such a sequence is referred to as a sequence of ones, i.e., each SM would include multiple sequences of ones. Finally, the 19th SM was created by setting each sample to 1 if all other SMs were 0 for that sample.

Due to this conversion to an SM, the start and end indices for each sequence of ones for a given SM indicate a fiducial point. Thus, for each fiducial point in the systolic complex, the sequences of ones for five SMs (not counting the 19th SM) either started or ended at that particular fiducial point, while this was so for two SMs in the diastolic complex. Additionally, note that the Cs-Ls and Cd-Ed interval definitions constituted SMs for identifying the full systolic complex and diastolic complex, respectively. The SMs resulted in the complete semantic segmentation of heart sounds and the 11 fiducial points.

Afterward, the SCG signals and corresponding SMs were divided into segments of 10 s to ensure a fixed algorithm length, and this was deemed a sufficient length to include the temporary and cyclic information in the window. An overlap of 2 s was introduced in the windowing to ensure that all the beats would appear in their full length at least once. If a beat was not represented in its full length due to the windowing, the whole beat was removed from that 10-s SM to ensure that the algorithm was presented with complete beats. In every 10-s segment, the SCG signal was min–max normalized, so the amplitudes assumed values between 0 and 1.

### 2.6 The U-Net architecture used for semantic segmentation

**Figure 3:**
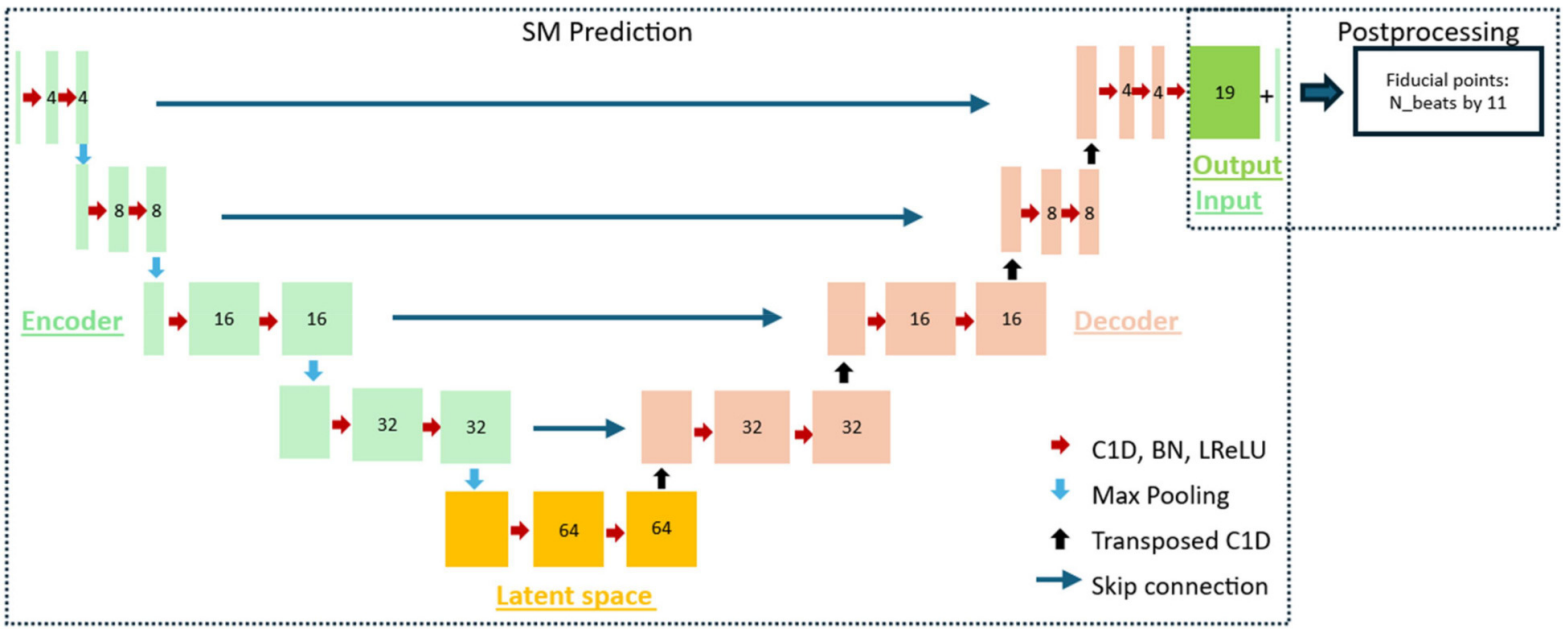
The architecture of the algorithm used for the delineation of the fiducial points, consisting of the SM prediction part and the postprocessing part. The SM prediction part consisted of a U-Net with an encoder, a latent space, and a decoder. Each block consisted of a one-dimensional convolutional layer (C1D), batch normalization (BN), and a leaky ReLU activation layer (LReLU). Between the encoding blocks, max pooling was introduced to downsample the feature maps, while a transposed one-dimensional convolutional layer was introduced between the decoder blocks to upsample the feature maps. Additionally, skip connections were introduced between each level of the encoder–decoder block. The 19 SMs from the model and the input SCG were used in the postprocessing to detect the fiducial points.

The SeismoTracker consisted of two parts: semantic segmentation of SMs and postprocessing for fiducial point detection. As in similar semantic segmentation algorithms (24–26), a U-Net architecture originally described by Ronneberger et al. (31) was used. The U-Net architecture and its connection to the postprocessing are illustrated in Figure 3. The U-Net architecture was constructed to handle one-dimensional 10-s SCG input, while the output of the U-Net consisted of the 19 predicted SMs. Each sample in each SM consisted of the probability of the given sample being between the given two fiducials in the particular SM.

The U-Net was constructed using an encoder consisting of four blocks, a latent space with one block, and a decoder consisting of four blocks. Each of the nine blocks consisted of two subblocks, and these subblocks consisted of a one-dimensional convolutional layer, a batch normalization layer, and a LeakyReLU activation layer with a slope of 0.01 for negative input values. Between each block in the encoder, max pooling was introduced to downsample the feature maps towards the latent space. Between the latent space and the blocks in the decoder, a transposed one-dimensional convolutional layer was introduced to upsample the feature maps toward the output. Skip connections between each encoding and decoding layer were introduced to allow the U-Net to use the features extracted in encoding directly in the decoding layer and thus enhance the semantic segmentation.

There was one filter in the input; 4, 8, 16, and 32 in the encoding layers, respectively; 64 in the bottleneck; 32, 16, 8, and 4 in the decoding layers, respectively; and 19 in the output layer, each representing one SM. This number of filters was used since it best optimized the recognition of the inherent temporal patterns in SCG. Additionally, in each of the blocks, a dilation was applied to optimize the model's ability to recognize the temporal and repetitive patterns that are inherently present in the SCG signal. The dilation was set to 1 in the first encoding layer and doubled towards the bottleneck, ending at 16, and then halved in the decoding layer, once again ending at 1 in the last decoding layer. The Adam optimizer and a learning rate of 0.001 were used.

Binary cross-entropy loss was used in the training process to optimize the prediction of the SMs. The U-Net was trained for 100 epochs; however, the model with the lowest validation binary cross-entropy loss without the dispersion of training and validation losses was used for testing. This was at 14 epochs. The U-Net was developed and trained in Python using the PyTorch module.

### 2.7 Postprocessing of segmentation maps

The output of the U-Net was 19 SMs representing sequences of probabilities between 0 and 1 related to the given fiducial point intervals based on the SCG signal. Therefore, the start and end indices of these predicted SMs would be used for fiducial point timing identification. However, to account for uncertainties in the predictions and get well-defined sequence edges in the SMs to obtain the precise location of the fiducial points, postprocessing steps were performed.

The postprocessing of the SMs consisted of two steps: (1) filtering the SMs that constituted the full complexes (i.e., the Cs-Ls and Cd-Ed SMs) and (2) filtering the other SMs. The concepts behind postprocessing steps 1 and 2 are illustrated in Supplementary Figures S1, S2, respectively. The following is a description of the filtering process.

1. Filtering of complex SMs (*SM05: Cs–Ls*, *SM17: Cd–Ed*) and the SM indicating no event (*SM19: No Event*)

We applied max-binarization by assigning 1 to the maximum probability and 0 otherwise per sample across *SM05: Cs–Ls*, *SM17: Cd–Ed,* and *SM19: No Event.*

a. Adjacent sequences of ones separated by less than 30 ms for *SM05* and 15 ms for *SM17* were gathered by setting intermediate samples to 1.
b. Sequences of ones with a duration of less than 50 ms for *SM05* and 20 ms for *SM17* were removed (samples set to 0), since these sequences were not considered valid lengths for the systolic and diastolic complexes, respectively.
c. *SM19* was adjusted according to the filtered masks by setting any samples that were 1 in *SM05* and *SM17* to 0 in *SM19*, and 1 otherwise.

Thus, by following this process, the sequences of ones in the SMs representing the entire systolic and diastolic complexes were binarized. Then, the other SMs with sequences of ones within these were filtered.

1. Filtering of other SMs (all SMs except *SM05: Cs–Ls*, *SM17: Cd–Ed,* and *SM19: No Event*)
  a. In all the other SMs, samples were set to 0 wherever the filtered *SM19* was 1. Thus, sequences of ones only appeared within the identified systolic and diastolic locations.
  b. All the other SMs were binarized with a 0.5 threshold (≥0.5 → 1, otherwise 0).
  c. We restricted the number of sequences of ones to one within the heart sound SMs (*SM05* and *SM17*). If there was more than one sequence of ones within the interval of *SM05* for systolic SMs and *SM17* for diastolic SMs, the longest sequence of ones was used, as it had the highest probability of being the correct sequence of ones.

Thus, the SMs were filtered, and well-defined edges were obtained in order to identify the fiducial points from the SMs. Supplementary Figures S1, S2 illustrate the filtering in one beat; however, the methods were applied for both complexes and for the full signal.

### 2.8 Fiducial point detection from postprocessed segmentation maps

Using the filtered SMs and the fact that each sequence of ones in these SMs represented an interval between fiducial points, the fiducial point timings were identified. We will explain this algorithm through an example of the identification of the fiducial point Fs. The process for Fs for one beat is illustrated in Supplementary Figure S3.

1. All SMs that included Fs were identified, namely, SM02: Cs–Fs, SM06: Ds–Fs, SM10: Fs–Gs, SM11: Fs–Ks, and SM12: Fs–Ls.
2. We then identified whether the sequences of ones should start or end at Fs, which was at the end for SM02 and SM06 and at the start for SM10, SM11, and SM12.
3. For each of the five relevant SMs, the valley closest to the start or end of the sequences of ones were identified. Thus, this resulted in five candidate timings for Fs.
4. The candidate timing with the most occurrences in each cardiac cycle was used as the Fs fiducial point for the given cardiac cycle.

This process was repeated for the other fiducial points, with the relevant masks for the given fiducial point. Moreover, for fiducial points Cs, Fs, Ks, Cd, and Ed, a valley should be found, while for fiducial points Ds, Gs, Ls, and Dd a peak should be found. Peak/valley identification was performed using the Python package Scipy. Fiducial points Es and Bd were found by identifying the shoulder of the slope preceding the valley fiducial points Fs and Cd, respectively.

### 2.9 Performance metrics

The test subjects were split into the following two groups: subjects with CD and subjects without CD. To evaluate algorithm performance with respect to fiducial point detection, the positive predictive value (PPV) and sensitivity were calculated in both groups. The performance metrics were calculated on a subject basis and then averaged for all subjects. PPV was calculated by the following equation:

$$PPV=\frac{TP}{TP+FP},$$ (1)

while the sensitivity was calculated by this equation:

$$S=\frac{TP}{TP+FN},$$ (2)

where TP denotes the true positives, FP the false positives, and FN the false negatives.

If a predicted fiducial point was within 10 ms of a corresponding true fiducial point, it counted as a TP. A 10 ms error margin was accepted as it was deemed non-significant. If there was no corresponding true fiducial point within 10 ms of a predicted fiducial point, it counted as an FP. If there was no predicted fiducial point within 10 ms of a corresponding true fiducial point, it counted as an FN. For the PPV and sensitivity, the median, interquartile range (IQR), mean, and standard deviation were calculated for each fiducial point in each group.

Using the Kruskal–Wallis test, we tested whether there was a significant difference in the PPV and sensitivity in fiducial point detection between the no-CD group and the CD group. Moreover, we used the same test to determine whether the fiducial point detection PPV and sensitivity differed significantly between the systolic complex fiducial points and the diastolic complex fiducial points. The Kruskal–Wallis test was used because neither the PPV nor the sensitivity was normally distributed. The sensitivity and PPV were pooled in these tests and thus only one test for the different test outputs was required.

Additionally, the postprocessed SMs were evaluated using the same measures as described by Renna et al. (24), where the centers of the predicted SM sequences of ones were evaluated against the centers of the annotated SM sequences of ones using the same measures as described above for the fiducial points. The borders of the sequences of ones were indirectly evaluated in the fiducial point detection evaluation. Moreover, the sample-by-sample accuracy was calculated as the fraction of samples in which the predicted SM value corresponded to the annotated SM value.

The algorithm was also evaluated using the external open-source dataset “Combined measurement of ECG, Breathing and Seismocardiograms.” This dataset consisted of, among others, SCG and simultaneously recorded ECG data pre-, during, and post-listening to classical music. The pre- and post-recordings lasted for 5 min each, while the during recording lasted for 50 min (32). However, this dataset had no labeled data. Therefore, the Neurokit2 Python Toolbox (33) was used to identify the R-peaks in the ECG data. An R-peak with an acceptable window of 50 ms on each side was considered as reference timing for the Fs fiducial point, based on which the recall and sensitivity were calculated.

Additionally, the proposed algorithm was evaluated against a simple algorithm using peak detection and decision rules, namely, the open-source PulsatioMech MATLAB toolbox (34). This algorithm only detected the fiducial points corresponding to Es, Fs, and Gs, which is why the evaluation was only conducted based on these fiducial points in the test dataset in this study. The Wilcoxon signed rank test was used to test for differences in specificity and recall between the two groups when using the two different algorithms. The Wilcoxon signed rank test was used as this was a paired observation of the two algorithms using non-parametric data.

In order to examine the explainability of the U-Net prediction model, the Grad-CAM visual explanation method (35) was used for segmentation maps SM05, SM11, SM17, and SM19. These examples can be found in Supplementary Figure S7.

### 2.10 Computational efficiency

All the data in this study's database were processed using the SeismoTracker algorithm. This data corresponded to 40,901 seconds of SCG signals. The data were processed using an Intel® Core™ i9–14900K CPU. The 40,901 seconds of SCG signals were processed in a total time of 141.4 s, corresponding to a throughput of 290 s, i.e., the CPU processed 290 s of SCG signals in 1 s, proving the algorithms’ feasibility for real-time dependent wearables.

## 3 Results

A total of 212 patients were included in this study across the datasets. In total, 15 subjects were excluded, the reasons for which were described in the individual studies from which the data were acquired, resulting in 198 subjects included in this study. The demographics of the included subjects in the training, validation, and test splits across the no-CD and CD groups are shown in Table 1. The algorithm was developed and trained using data from 136 subjects, with a total of 28,927 individual beats, and was tested using data from 42 subjects, with a total of 9,821 individual beats.

**Table 1:** Demographic data of the subjects in training, validation, and test groups within the no-CD and CD groups.

Variable	No-CD	CD	*p*-Value
Training			
* N*	119	17	
* *Age (±std)	35.4 (12.5)	55.1 (17.3)	0.000
* *Male/female	57/64	13/2	
* *Number of beats	23,647	5,280	
* *Mean HR (±std)	60.7 (9.8)	65.3 (7.2)	0.096
Validation			
* N*	17	3	
* *Age (±std)	38.2 (13.3)	59.3 (7.3)	0.021
* *Male/female	8/9	3/0	
* *Number of beats	3,112	592	
* *Mean HR (±std)	56.8 (11.6)	59.9 (15.0)	0.708
Test			
* N*	34	8	
* *Age (±std)	36.5 (14.1)	50.5 (16.5)	0.022
* *Male/female	13/21	8/0	
* *Number of beats	6,464	3,357	
* *Mean HR (±std)	60.0 (10.1)	55.3 (10.4)	0.253

**Figure 4:**
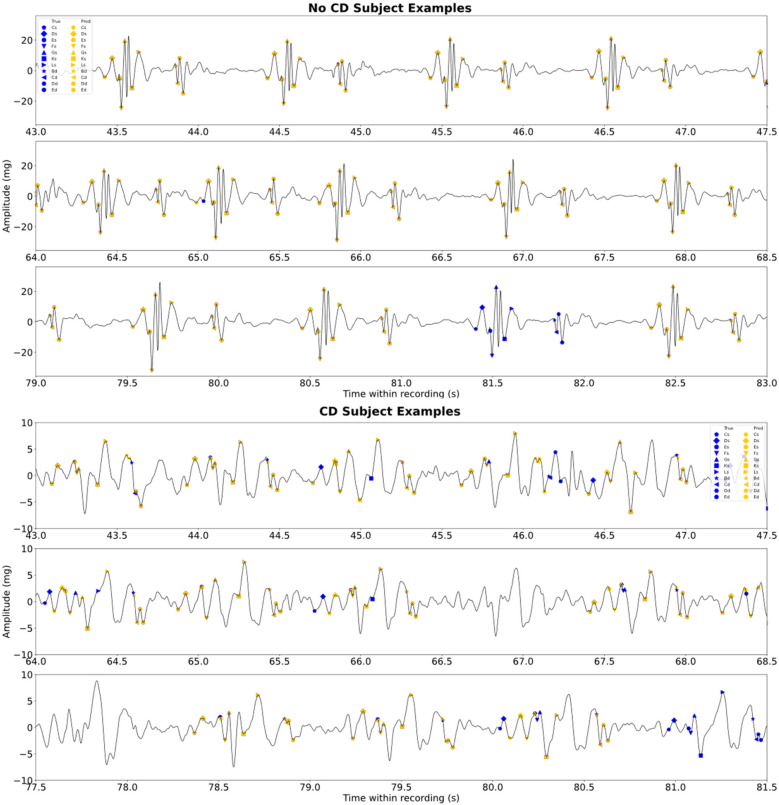
Examples of the sequences of true and predicted fiducial points for a test subject without CD and a subject with CD. The subject with CD is a sequence without pacing. Note that there are blue scatters behind the orange scatters. The figure illustrates some of the error types experienced.

Examples of sequences of true and predicted fiducials are illustrated in Figure 4 for a subject without CD and a subject with CD, respectively. The CD subject originated from a study on subjects with biventricular pacemakers that used a recording sequence without pacing (14).

**Figure 5:**
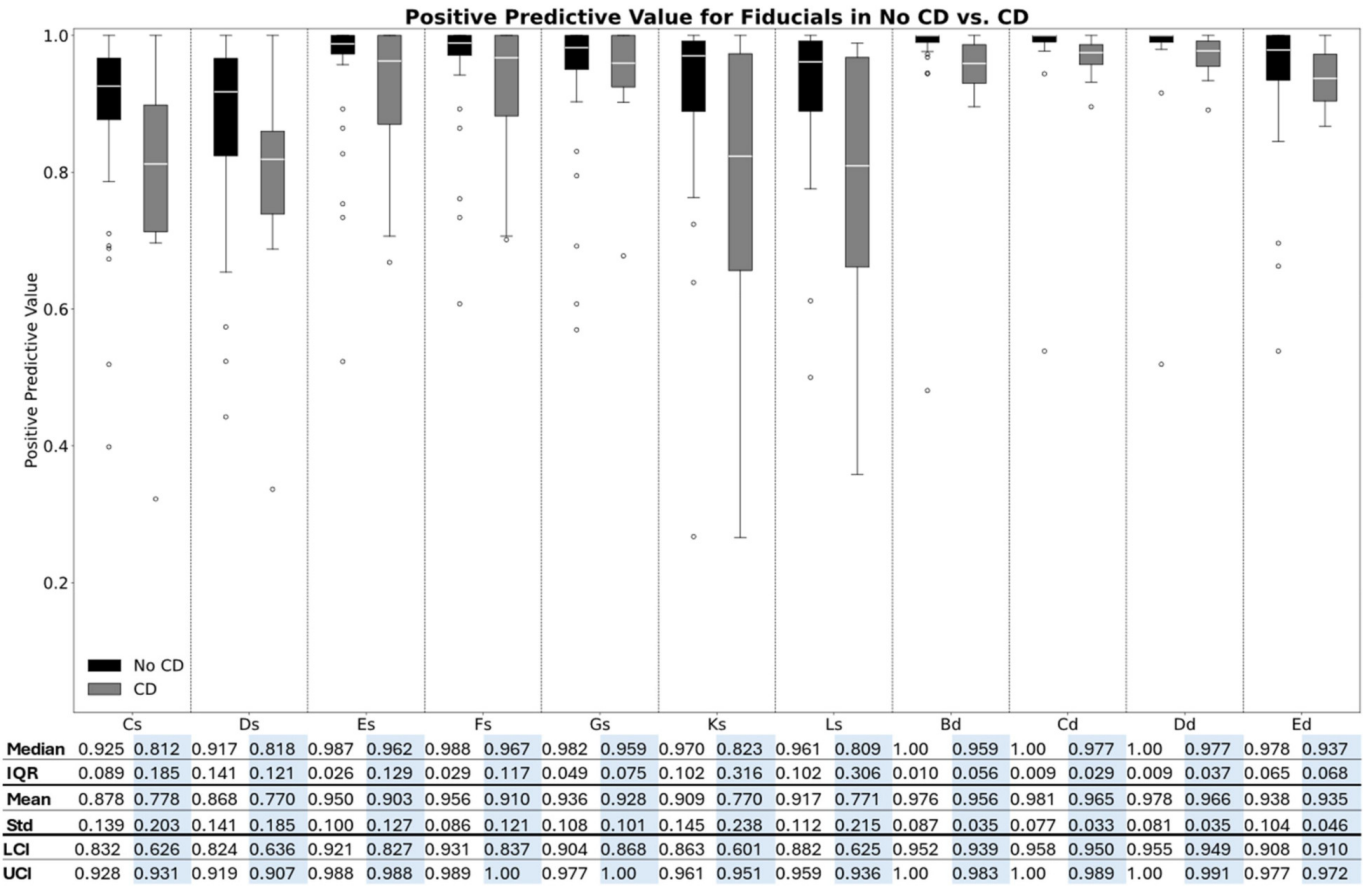
Box plot of the positive predictive values for each fiducial point in the two groups**.**

**Figure 6:**
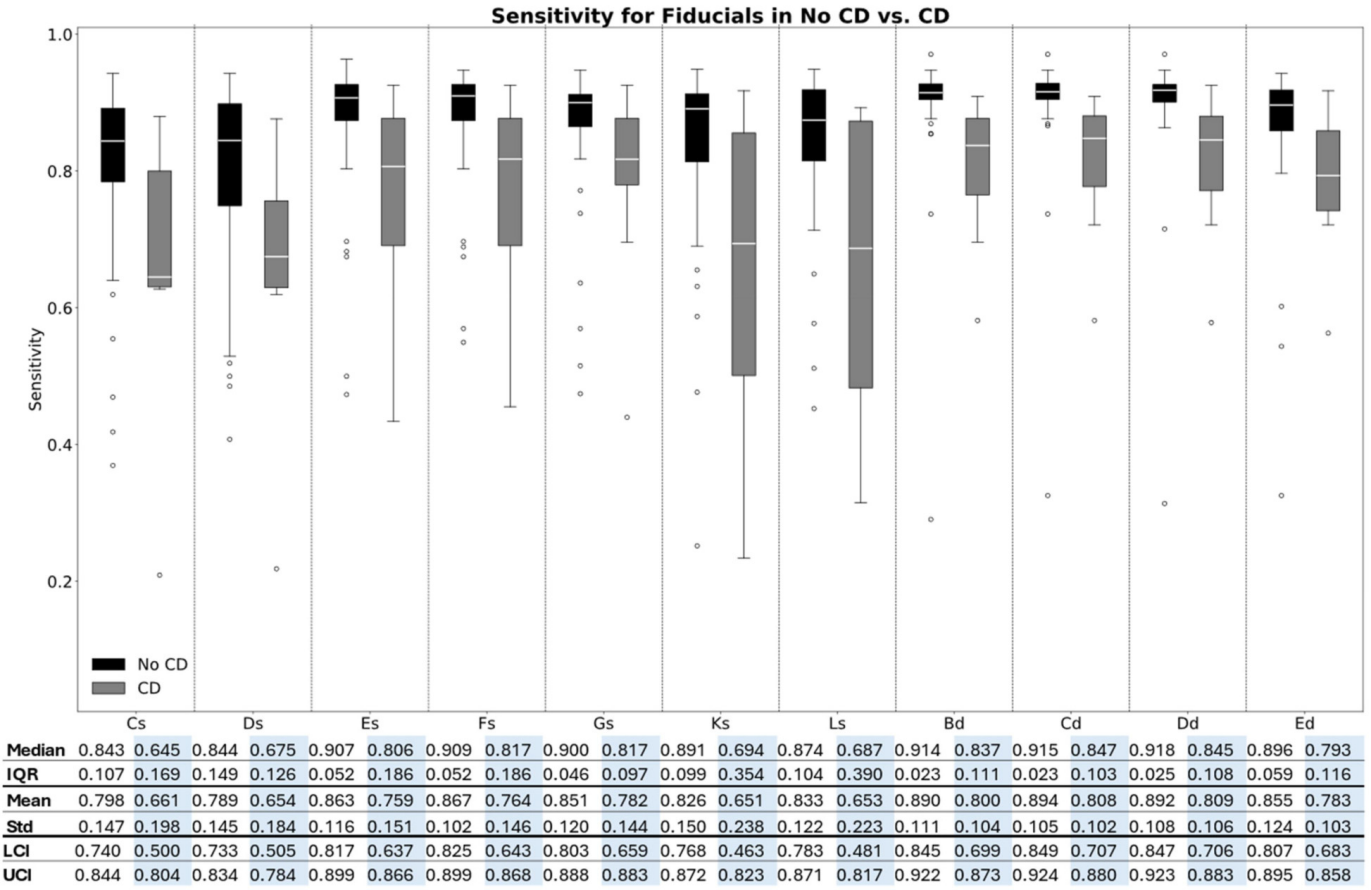
Box plot of the sensitivity for each fiducial point in the two groups**.**

The PPV and sensitivity for each fiducial point in the two groups are illustrated in box plots in Figures 5, 6, respectively. Moreover, the median, IQR, mean, and STD for each fiducial point in each group are highlighted in Figure 5 for PPV and Figure 6 for sensitivity.

As illustrated in Figures 5, 6, the median PPV and sensitivity were generally greater in the no-CD group compared to the CD group, while the IQRs for both precision and recall were generally narrower in the no-CD group compared to the CD group. Additionally, the Kruskal–Wallis test indicated a significant difference in the PPV (*p* < 0.001) and sensitivity (*p* < 0.001) between the two groups for all fiducial points.

Additionally, as Figures 5, 6 highlight, the PPV and sensitivity in both groups were generally higher for the diastolic complex fiducial points (Bd, Cd, Dd, and Ed) compared to the systolic complex fiducial points (Cs, Ds, Es, Fs, Gs, Ks, and Ls). The Kruskal–Wallis test indicated a significant difference in the PPV (*p* < 0.001) and sensitivity (*p* < 0.001) between the systolic and diastolic complex fiducial points.

The fiducial points Es, Fs, and Gs resulted in a median PPV (IQR) of 0.987 (0.026), 0.988 (0.029), and 0.967 (0.049), respectively, and in a median sensitivity (IQR) of 0.907 (0.052), 0.909 (0.052), and 0.900 (0.046), respectively, in subjects with no CD. In comparison, Cs, Ds, Ks, and Ls generally resulted in a lower PPV and higher PPV-IQR of 0.925 (0.089), 0.917 (0.141), 0.970 (0.102), and 0.961 (0.102), respectively, in subjects without CD and in a lower median sensitivity and higher sensitivity-IQR of 0.843 (0.107), 0.844 (0.149), 0.891 (0.099), and 0.874 (0.104), respectively, in subjects without CD.

The highest obtained median PPV was 1.00 for the Bd, Cd, and Dd fiducial points in the no-CD group, while these fiducial points also accounted for the highest median sensitivities of 0.914, 0.915, and 0.918, respectively, in the no-CD group. The highest obtained median PPV in the systolic complex was 0.988 for Fs in the no-CD group and Fs also resulted in the highest sensitivity of 0.909.

For all fiducial points, both the PPV and sensitivity were generally higher in the group without CD compared to the group with CD. Moreover, the dispersion was generally higher within the CD group for both the PPV and the sensitivity. The boxplots for the accuracy, PPV, and sensitivity of the predicted SMs are shown in Supplementary Figures S4–S6, respectively.

The evaluation on the external dataset yielded a median PPV for the pre-, during, and post-intervention periods of 0.914 (0.047), 0.874 (0.088), and 0.914 (0.047), respectively. The sensitivity was 0.664 (0.396), 0.620 (0.439), and 0.611 (0.396) for the pre-, during, and post-intervention periods, respectively.

The PulsatioMech fiducial point detector resulted in a median PPV of 0.494 (0.560) and a median sensitivity of 0.503 (0.562) for the healthy group. The PPV and sensitivity of SeismoTracker were 0.989 (0.025) and 0.905 (0.051), respectively, for the fiducial points Es, Fs, and Gs in the group with no CD. PulsatioMech resulted in a median PPV of 0.782 (0.419) and a median sensitivity of 0.671 (0.418) in the CD group, while SeismoTracker resulted in a median PPV of 0.965 (0.098) and a median sensitivity of 0.795 (0.169). The results for each fiducial point for each algorithm in both groups are illustrated in Supplementary Figure S8. The Wilcoxon signed rank test indicated a significant difference in the PPV and sensitivity (*p* < 0.001) between the two algorithms in the no-CD group. The difference was significant for the PPV (*p* = 0.015) in the CD group, while the difference in sensitivity in the CD group was not significant (*p* = 0.109).

## 4 Discussion

The deep-learning algorithm developed in this study is the first automatic algorithm to detect a high number of fiducial points in SCG and to be validated on tens of thousands of cardiac cycles from hundreds of subjects. Previous studies have automatically detected up to four fiducial points or locations of the systolic and diastolic complexes on a beat-to-beat basis in either SCG or the similar PCG signal (16, 17, 20–25). The fiducial points typically detected in other algorithms with a similar purpose are the points corresponding to Es, Fs, Gs, and Cd (16, 20, 23), which are also the points that resulted in a high PPV and sensitivity in this study. However, besides these fiducial points, this algorithm also detected further fiducial points with a similarly high PPV and sensitivity.

The 11 fiducial points automatically detected in this study have been related to specific physiological events in the cardiac cycle, such as the timing of valve opening and closing and peak blood flow in the atria and ventricles (6). These fiducial points have also been used in many different settings, such as cardiorespiratory fitness (12), correlation with preload changes (10, 11), possible assessment of biventricular pacing (14), and electromechanical coupling (9).

Since SeismoTracker performed well on a dataset that also included subjects with CD and recordings with modulations of cardiac mechanics, such as the tilt experiment and the saline infusion, it has the potential to be adapted to many different SCG morphologies. The ability to track such changes automatically promises to be a powerful tool for the continuous monitoring of myocardial mechanics and hemodynamic status. Additionally, apart from slopes and amplitudes, the morphology of SCG between subjects with CD and non-CD subjects is different, and the algorithm also has the ability to recognize these patterns. Therefore, the approach has the potential to provide an easy-to-use and extensive evaluation of myocardial performance, with clinical use cases such as the detection of cardiac dysfunction, change in heart failure status, and cardiac arrhythmias.

Other studies that proposed algorithms for fiducial point detection were based on prior knowledge, decision rules, and thresholds (17, 20, 21). The algorithms in these studies had good performance; however, the algorithms were either evaluated on a limited number of subjects, requiring the tweaking of parameters and thresholds, or needed ECG R-peak gating for optimal performance. The algorithm proposed in this study is an SCG standalone algorithm that detects more fiducial points than previous algorithms and was evaluated in subjects with and without CD. Moreover, while retraining would be required to adjust for other CD SCG morphologies, this should be possible considering the performance of the SeismoTracker in subjects with CD. Additionally, the SeismoTracker outperformed the simple decision-rule and peak detection algorithm in the fiducial points Es, Fs, and Gs. In addition to this, the SeismoTracker identifies 11 fiducial points, while the PulsatioMech only detects three. However, the difference between these algorithms was not statistically significant in the no-CD group, underlining the necessity for more subjects with CD in the algorithm. However, the PulsatioMech does not implement signal quality control, which is inherently implemented in the SeismoTracker. Additionally, the IQR for PulsatioMech’s PPV and sensitivity was high, indicating that it performed well for some subjects and poorly for others. Additionally, PulsatioMech is based on a maximal peak identification, and it is not always the peak with the largest amplitude that is the fiducial point, resulting in lower performance in this algorithm.

Other studies have proposed deep learning methods for the segmentation of the systolic and diastolic complexes in SCG or PCG (24, 25), with performance comparable to or better than that in this study. However, this study included more subjects and subjects with different types of CD, covering a wide variety of SCG signals, and also estimated more fiducial points. The morphology of the SCG signal is inconsistent across gender, age, weight, and the presence of CD, while the variation in morphology additionally varies in each cardiac cycle, which is also challenging for the algorithm. Despite this, the proposed algorithm resulted in a relatively high PPV and sensitivity across the fiducial points, proving the concept of using a deep learning model for beat-to-beat estimation of multiple fiducial points.

There was a significant difference in the PPV and sensitivity for fiducial point detection between the two groups, indicating that the model was more efficient in the subjects with no known CD compared to the subjects with known CD. This could be caused by multiple factors. First, the algorithm was trained on a relatively low number of subjects with CD, which naturally would result in lower performance in the algorithm evaluation. Moreover, the SCG morphology in some of the subjects was very irregular and different from the subjects without CD, especially in the recordings from the cardiac resynchronization therapy study that had the patients’ biventricular pacemakers switched off, resulting in lower performance. Despite this, the PPV and sensitivity obtained across the different fiducial points were still high, which indicates that the algorithm can even recognize patterns in highly irregular SCGs. This should be investigated in future studies using the algorithm for the classification of the cardiac diseases present in this study's database.

The median PPV and sensitivity were significantly higher for the fiducial points in the diastolic complex (Bd, Cd, Dd, and Ed) compared to the fiducial points in the systolic complex (Cs, Ds, Es, Fs, Gs, Ks, and Ls). These findings indicate that there are consistent patterns in the diastolic complex as the U-Net successfully recognizes and can be applied to unseen data, even though respiration causes amplitude modulation in the diastolic complex (36, 37). However, they also indicate that these modulations are relatively regular and thus relatively simple for the algorithm to recognize, which underlines the strengths of this type of algorithm.

Additionally, in the systolic complex, the fiducial points Es, Fs, and Gs, also referred to as the W complex (20), had the highest median sensitivity and PPV and smallest PPV-IQR and sensitivity-IQR compared to the other systolic complex fiducial points, namely, Cs, Ds, Ks, and Ls. This could be caused by the beat-to-beat inconsistency in SCG around these points. The inconsistency may not be as regular as the amplitude modulation caused by respiration in the diastolic complex. Thus, these points were also more challenging for the annotator to identify without ECG, i.e., it would also be challenging for the algorithm to identify. Moreover, this indicates that the mechanics occurring in atrial systole and peak systolic outflow seem to be more irregular beat-to-beat, while the peaks around the valve openings and closings seem more consistent on a beat-to-beat basis. Finally, the differences in the median PPV and sensitivity between the non-CD group and CD groups for these fiducial points were larger than for the other fiducial points. This may indicate that the atrial systole and peak systolic outflow are even more irregular and variant on a beat-to-beat basis in subjects with CD compared to non-CD subjects.

## 4.1 Study limitations

Even though the study included many subjects with many individual cardiac beats, the number of subjects with CD was still relatively low in the context of deep learning. Thus, increasing the number of subjects and types of CD would enhance the algorithm's performance, especially when using this data-driven model. Moreover, including more subjects with additional types of CD could enhance the algorithm's performance in subjects with CD, thus expanding the model’s applications. Additionally, in the proposed annotation pipeline, it would be easy to include and annotate more SCGs to enhance the data-driven algorithm.

The proposed algorithm was able to reliably detect the Fs fiducial point in the external dataset. However, the comparison may not be fully representative of the algorithm’s performance. First, the R-peak was easily identifiable despite the influence of movement; however, in this case, the SCG signal was noisy, and no fiducial points were thus detected. Additionally, the R-peak detection algorithm may not always be accurate. Both aspects would affect the results, even though the validation using the external dataset supports the generalizability of the algorithm.

In addition, the mean heart rate in the training set was 60–65 beats per minute, and the measures were performed in a relaxed setting. Therefore, it would be reasonable to include signal sequences with higher heart rates and more heart rate variability within subjects to increase the representability of the algorithm.

The annotation in this study was different from that in other studies in this field and the risk of introducing bias when using the algorithm is present. To reduce bias, the annotation rules were strictly followed independently of the initial annotations, and the annotation was guided by concurrent ECG. Moreover, the annotator did not know if the given subject was a training, validation, or test subject. Thus, there was no bias related to not modifying the initial annotations in the test set to obtain a better model performance. However, when using this method, a very large number of individual SCG beats were annotated relatively fast, which proves the scalability of the method, which is necessary when developing such data-driven models.

Moreover, the SeismoTracker algorithm is still quite dependent on a relatively high sample rate, resulting in high hardware requirements for wearable integration. Moreover, the algorithm has not been implemented for real-time use, and all data are processed offline after data acquisition. To be used in a wearable device, SeismoTracker still needs adjustments for real-time processing.

## 5 Conclusion

An algorithm for the automatic detection of 11 fiducial points in SCG using a U-Net and simple postprocessing methods was developed and tested in subjects with and without CD, with excellent performance, proving that this data-driven algorithm can adapt to different SCG morphologies without adjusting and refining decision-rule-based algorithms that depend on prior expert knowledge.

## Data Availability

The datasets presented in this article are not readily available as it is private data and therefore under a closed license. Requests to access the datasets should be directed to the corresponding author.
