# Supplementary material for: Deep learning-based beat-to-beat delineation of heart sounds and fiducial points in seismocardiography
Source: Front Digit Health. 2025 Dec 4;7:1699611. doi: 10.3389/fdgth.2025.1699611 (PMC12712800; doi:10.3389/fdgth.2025.1699611)
Supplement: Supplementary file 1 [file Datasheet1.pdf]

Supplementary Materials

Figure S1: Step 1 of Filtering Predicted SMs

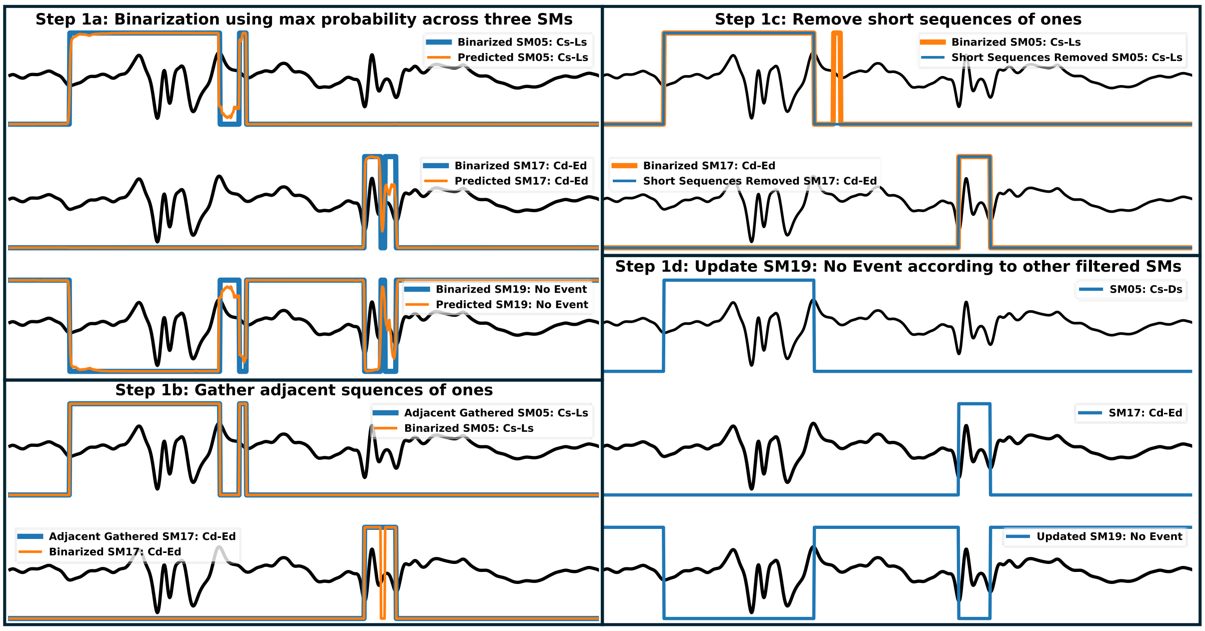

Figure S1: An illustration of the postprocessing steps for the SMs; SM05: Cs-Ls, SM17: Cd-Ed, and SM19: None. The illustration is just for one heartbeat, but the process is performed on each beat in the recording. Firstly, the three masks are binarized. Secondly, adjacent sequences are gathered. Thirdly, short sequences not adjacent are removed. Lastly, the SM19: None SM is updated according to the filtered SM05: Cs-Ls and SM17: Cd-Ed.

Figure S2: Step 2 of Filtering Predicted SMs

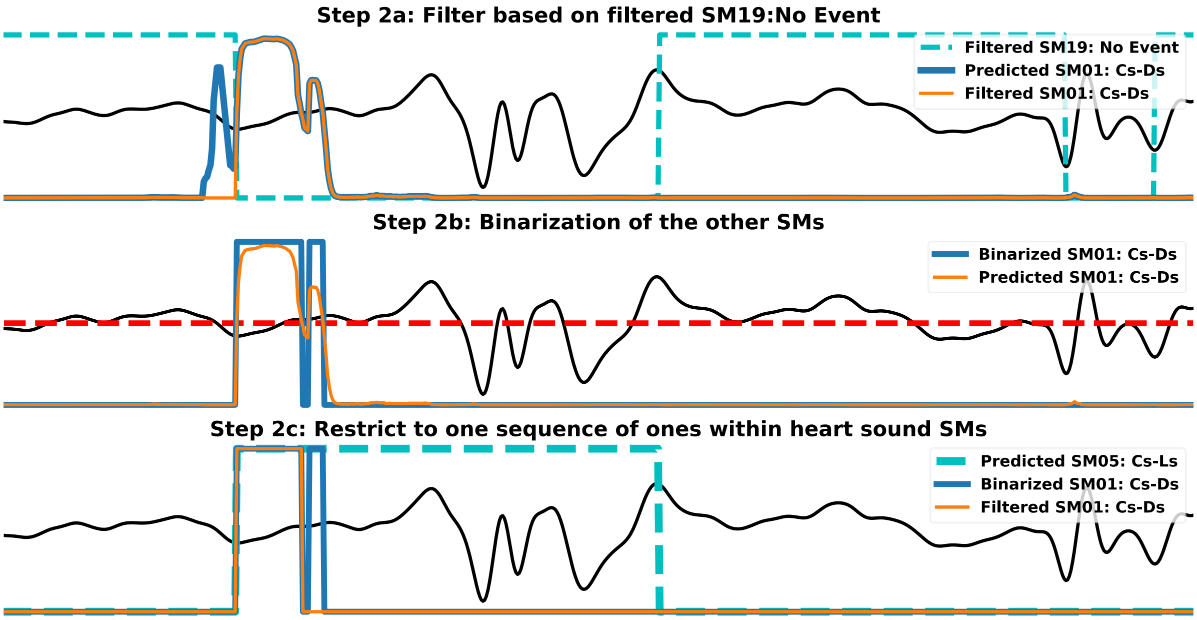

Figure S2: An illustration of the post processing of the SMs that do not cover either the full systolic complex or the full diastolic complex. The example is based on a filtering process of SM01: Cs-Ds. The approach is the same for the other SMs that are not SM05: Cs-Ls, SM17: Cd-Ed, and SM19: No Event. Firstly, the SM is filtered based on the filtering of step 1 to ensure that no SMs would assume the value of 1, where the filtered SM19: No Event assumes 1. Secondly, the SM is

binarized by thresholding. Lastly, it is ensured that only one sequence of ones appear within the given heart sound. In this case, it is ensured that there only is one sequence of ones of SM01: Cs-Ds within SM05: Cs-Ls.

Figure S3: From filtered SMs to Fiducial Point Detection

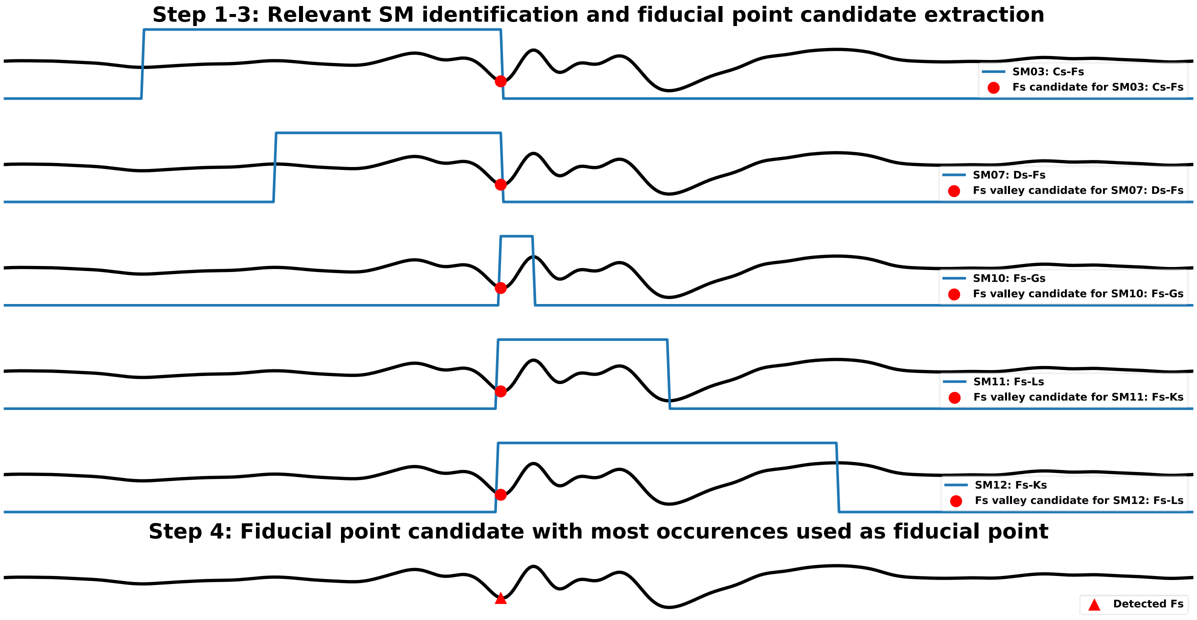

Figure S3: The SM-postprocessing resulting in fiducial point detection. The process is based on one beat for detecting valley fiducial point Fs. Firstly, the relevant SMs are extracted. Secondly, it is identified whether the given fiducial occurs at the start or end index of each identified SM. Thirdly, the valley closest to start or end of the given sequence of ones is identified. The valley with most occurrences is used as given fiducial point. If it is peak fiducial point, the peak would be found instead of a valley.

Figure S4: SM Based Accuracy

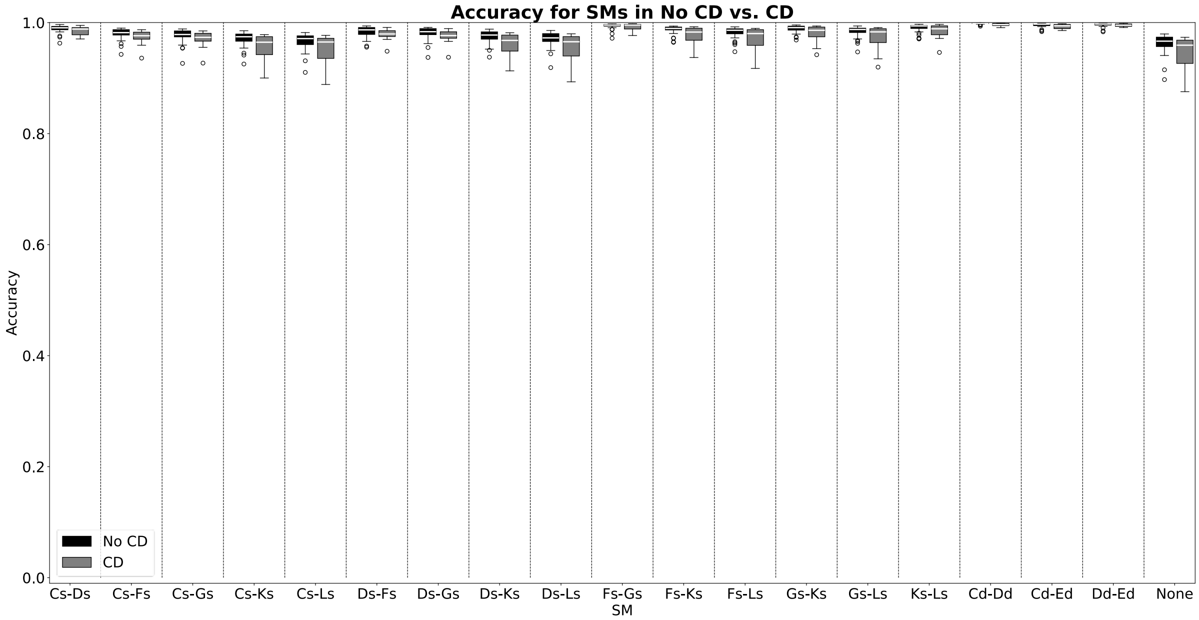

Figure S4: Accuracy of the center of the predicted and filtered SMs compared to the true SMs.

Figure S5: SM based Positive Predictive Value

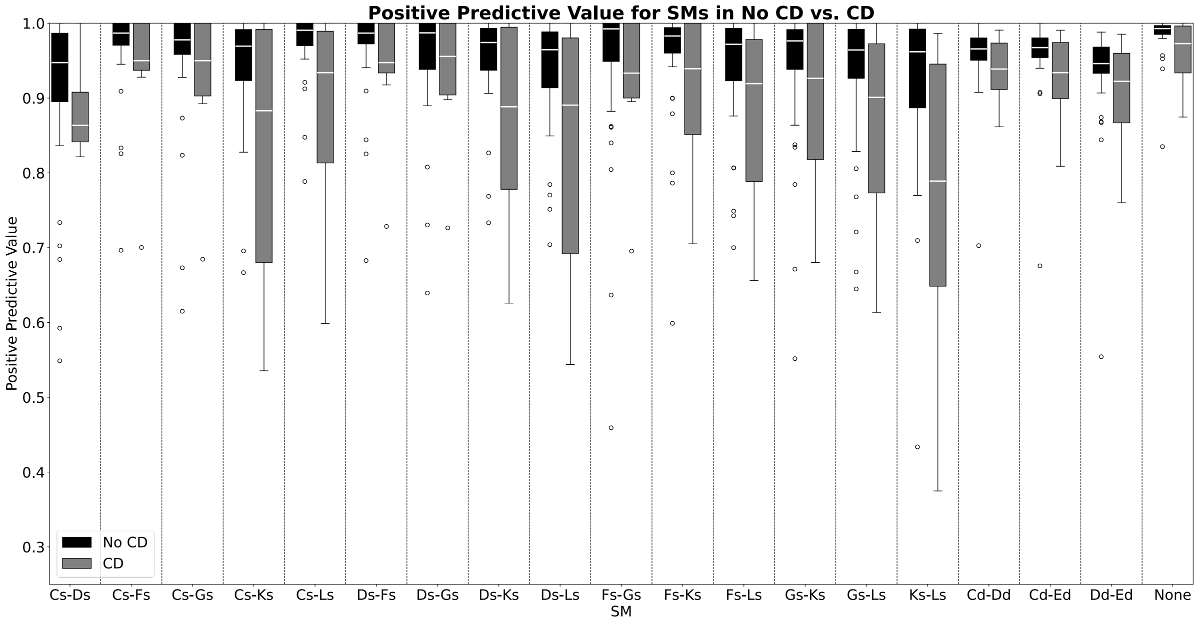

Figure S5: Positive predictive value of the center of the predicted and filtered SMs compared to the true SMs.

Figure S6: SM Based Sensitivity

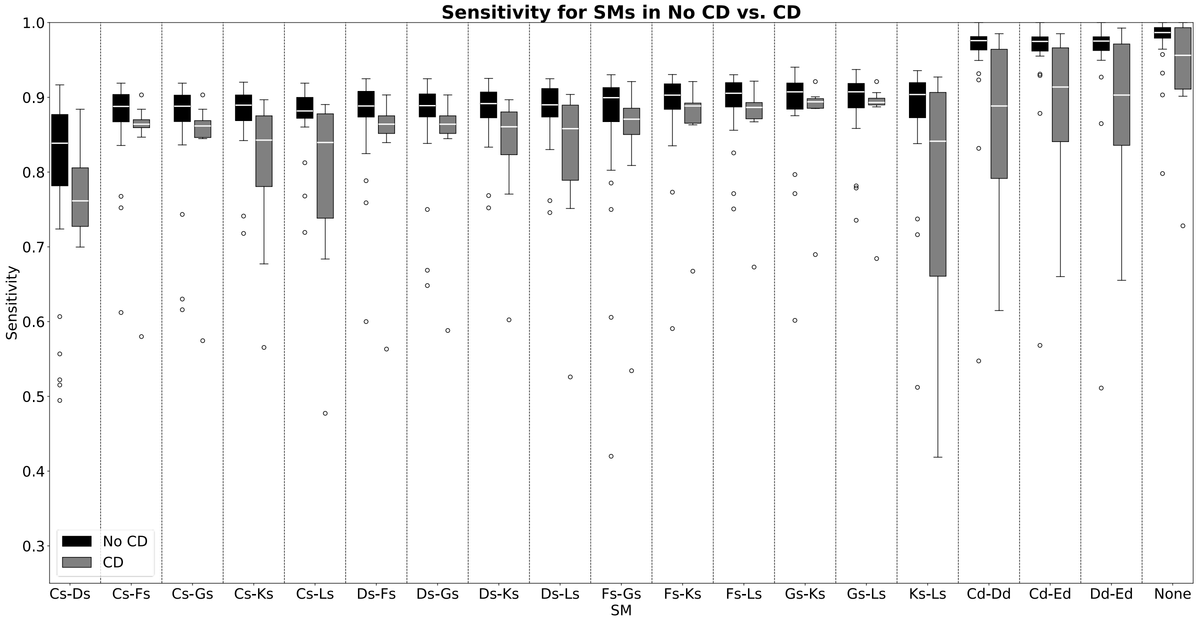

Figure S6: Positive predictive value of the center of the predicted and filtered SMs compared to the true SMs.

Figure S7: Aligned Saliency Maps

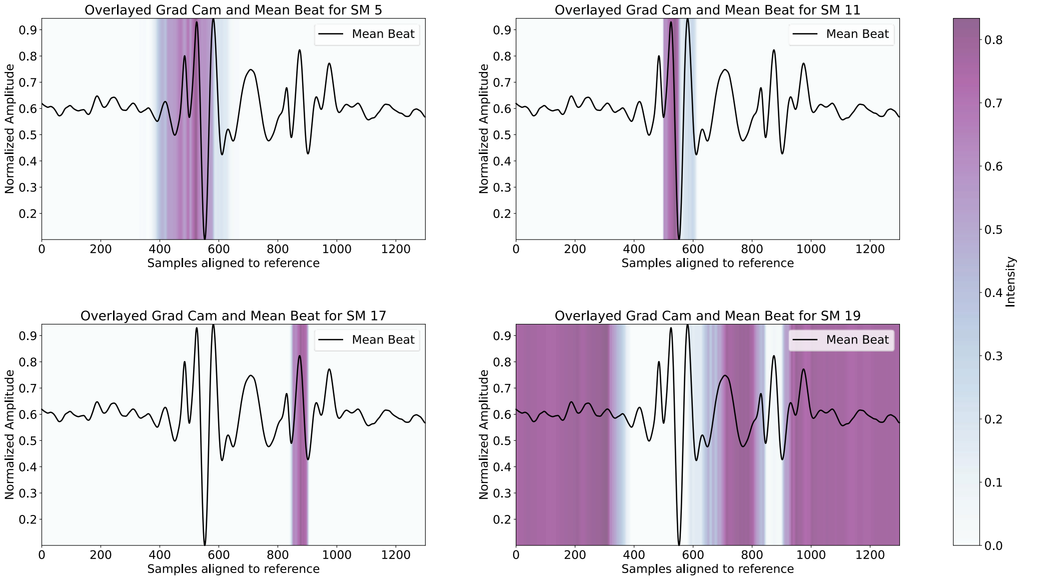

Figure S7: The beat-aligned Grad-CAM intensities for an SCG recording for the segmentations maps SM05, SM11, SM17, and SM19. All indicating that the algorithm focuses on the correct parts of the input signals for making its predictions.

Figure S8: Comparative Evaluation

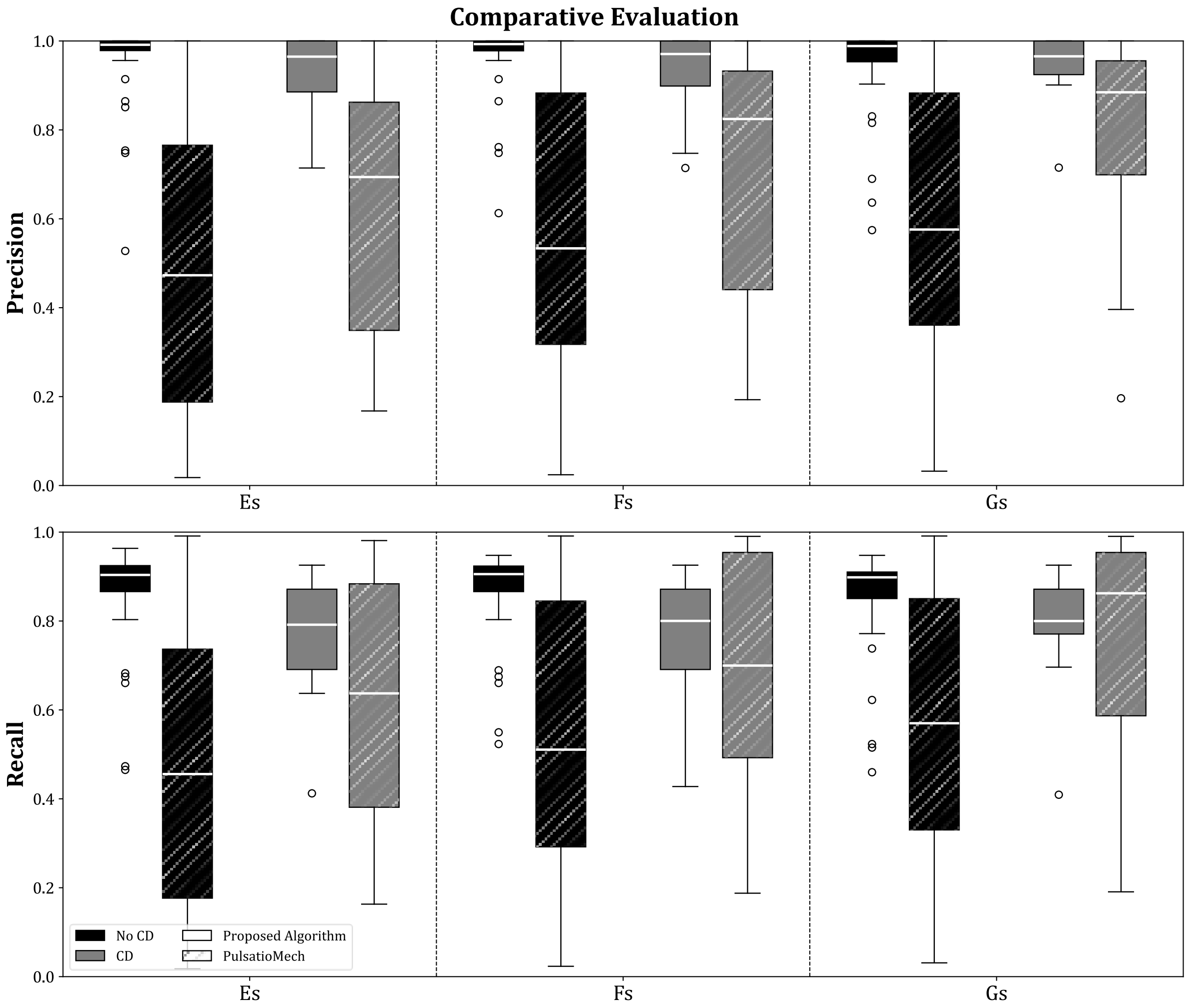

Figure S8: The PPV and sensitivity of the fiducial points Es, Fs, and Gs for both algorithms for both the subjects with no CD and the subjects with Cd.
